# Beyond the Diagnosis: A Journey of an 8-Year-Old Girl with Patau Syndrome: Case Report

**DOI:** 10.3390/children12121632

**Published:** 2025-12-01

**Authors:** Natalia Aleksander, Adrian Bukała, Wiktoria Borowska, Katarzyna Czapla, Krzysztof Bylok, Mikołaj Magiera, Tomasz Czerwiec, Krystyna Stencel-Gabriel

**Affiliations:** 1 Student Scientific Society at the Chair and Clinical Department of Pediatrics, Hospital No. 2 Bytom, School of Health Sciences, Medical University of Silesia in Katowice, 40-055 Katowice, Poland; 2 Department of Paediatrics, Faculty of Medical Sciences in Katowice, Medical University of Silesia, 40-055 Katowice, Poland

**Keywords:** Patau syndrome, trisomy 13, pediatric palliative care, cytogenetic diagnosis in utero, omphalocele, gastrostomy, enteral nutrition

## Abstract

**Background/Objectives**: Patau syndrome (trisomy 13) is a rare genetic disorder with high mortality, and poor prognosis. Patients surviving beyond infancy usually present with severe psychomotor delays, failure to thrive, intellectual disabilities and seizures. Female sex and mosaic trisomy 13 are considered positive prognostic factors. **Methods**: Here we report an 8-year-old female patient with Patau syndrome, diagnosed prenatally, born prematurely at 35 + 4 weeks of gestation via vaginal delivery as a third child of 33 years old healthy and unrelated parents. The birth weight was 2087 g, Apgar scored 9 at 1 min and 10 at 5 min, also self-ventilating in room air since birth. The patient has several associated congenital abnormalities; however, medical adjustments such as multiple surgeries, PEG, hearing aids, glasses, anti-epileptic medications, and suction support the girl’s daily life. The patient attends a primary school with specialist support that fosters her physical and sensory development and promotes progress in communication. Despite the numerous obstacles she faces, the girl’s journey demonstrates remarkable growth and development with the support of an interdisciplinary care team. It highlights the critical role of personalized care and early intervention. **Conclusions**: Due to the increasing survival rates of patients with Patau syndrome, complex and multidisciplinary care is required for both the patients and their families to achieve the best possible outcomes and ensure proper care, growth, and development of the child. All medical procedures must be thoroughly assessed for potential complications and viable improvement in quality of life.

## 1. Introduction

Patau Syndrome (trisomy 13) is a rare genetic disorder caused by the presence of an additional copy of chromosome 13 in all or some body cells. This chromosomal abnormality disrupts normal embryonic development, resulting in multiple congenital anomalies. It is characterized by severe malformations including cleft lip and/or palate, cerebral defects such as microcephaly or holoprosencephaly, congenital heart disease, anophthalmia or microphthalmia, simian crease, postaxial polydactyly, trigger thumbs, and capillary hemangiomas [1].

Trisomy 13 is the third most common autosomal trisomy, following Down syndrome (trisomy 21) and Edwards syndrome (trisomy 18), and is associated with a high rate of fetal and neonatal morbidity and mortality [2]. The estimated incidence is approximately 1 in 10,000–20,000 live births, with the majority of affected fetuses dying in utero [1]. The median survival time for live-born infants with full Trisomy 13 is between 7 and 10 days in the absence of medical intervention. Over 90% of affected neonates die within the first year of life [1,2,3,4,5]. Risk factors for early death include male sex, preterm delivery, and the presence of congenital heart defects. The most frequent causes of death are heart failure and central apnea [6,7]. However, survival beyond the first years has been documented, particularly in cases with mosaic Trisomy 13, female sex, the absence of lethal congenital malformations, and when aggressive medical or surgical interventions are implemented [8,9,10,11].

Historically, Patau syndrome was considered a universally fatal diagnosis, with palliative care typically recommended as the primary management strategy. In recent years, this paradigm has been increasingly challenged. New studies emphasize that terms such as “incompatible with life” or “lethal” are outdated and fail to reflect the variability in clinical outcomes. Consequently, there is growing recognition of the need for individualized care plans and reassessment of treatment strategies.

This paper aims to present the case of an 8-year-old female with Patau syndrome, illustrating long-term survival in individuals with this condition and emphasizing the role of an interdisciplinary approach in influencing both developmental outcomes and the clinical course of the disease.

## 2. Case Report

### 2.1. Fetal and Neonatal Period

The pregnancy was managed in the United Kingdom, where all pregnancies were offered screening for trisomies 21, 18 and 13 as part of the Fetal Anomaly Screening Progamme. Screening included first-trimester biochemical tests combined with ultrasound as well as a second-trimester anomaly scan [12]. During the first trimester, ultrasound was performed to measure nuchal translucency between 11 + 2 and 14 + 1 weeks of gestation, with a crown-rump length. First-trimester screening revealed decreased PAPP-A levels and ultrasound findings of increased nuchal translucency at 12th week of gestation, indicating an increased risk of chromosomal abnormalities, particularly trisomy 13, trisomy 18 and trisomy 21, therefore the pregnancy was classified as high-risk. Pregnancies identified as high-risk were offered invasive diagnostic testing, and in our patient prenatal diagnosis of Patau syndrome was established through amniocentesis performed at the 21st week of gestation, which revealed a non-mosaic chromosomal abnormality of 47, XX, +13 resulting from meiotic nondisjunction. Parents were counseled antenatally about the condition and life expectancy prior to delivery, further obstetric care was coordinated by a multidisciplinary team comprising specialists in obstetrics, fetal medicine, genetics, and neonatology. All procedures required adherence to informed consent protocols, provision of patient information, and documentation of the patient’s decision.

The patient was born prematurely via vaginal delivery at 35 + 4 weeks of gestation as a third child of a 33-year-old mother and a 33-year-old father. The parents were not related, and the older children were healthy. The mother had taken paracetamol and sumatriptan for migraine attacks and had no history of infectious diseases during pregnancy. The birth weight was 2087 g, the head circumference 31.0 cm and APGAR scores were 9 at the first minute and 10 at the fifth minute. The infant cried at birth, required no resuscitation, and demonstrated spontaneous respiration, within the first minute of life. She was transferred to the neonatal unit, self-ventilating in room air and hemodynamically stable.

On admission, an omphalocele containing intestinal loops at the base of the umbilical cord was noted. Surgical repair of the omphalocele was performed on day 3 of life. Postoperatively, the patient required mechanical ventilation and was successfully extubated after two days, remaining stable thereafter. She also received five days of phototherapy for neonatal jaundice.

**Figure 1:**
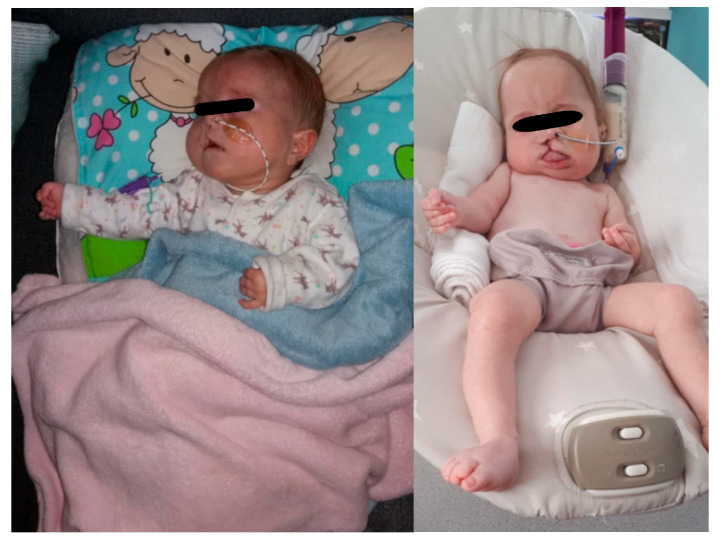
The female patient with Patau syndrome as an infant presenting phenotypic changes in Trisomy 13, including cleft lip, trigger thumbs and rocker bottom feet.

The newborn remained in the NICU for a total of 15 days, including 3 days in intensive care and 12 days in special care. During this period, multidisciplinary assessment was conducted. On physical examination, the patient exhibited several phenotypic features consistent with Patau syndrome, including cleft lip and palate, ocular hypertelorism, low-set ears, low anterior hairline, cutis aplasia, microcephaly, overlapping fingers, and rocker-bottom feet (Figure 1). No pulmonary, genital, renal abnormalities, or polydactyly were identified. Congenital heart defects, including ventricular septal defect (VSD), atrial septal defect (ASD), patent foramen ovale (PFO), and bicuspid aortic valve were identified; however, the girl remained hemodynamically stable and did not require cardiovascular support.

The patient presented no sucking reflex and hence parents were instructed in full NG feeding with expressed breast milk. After 15 days of hospitalization, the infant was discharged home in stable condition.

### 2.2. Early Life Period

At the age of 3 months, the patient developed myoclonic seizures. She has been receiving antiepileptic treatment since then (levetiracetam 200 mg twice daily and sodium valproate 120 mg twice daily). She has remained seizure-free for the past 3 years. Consequently, gradual weaning of sodium valproate is under consideration.

Since the age of 6 months, the patient has used bilateral hearing aids, although no recordable auditory brainstem responses (ABR) were detected. A cochlear implant has been recommended and remains under parental consideration. Ophthalmologic evaluation revealed anisometropic myopia and astigmatism, accompanied by constant right-sided exotropia. She has worn corrective glasses since the age of 2 and tolerates them well. She is able to visually track a light-up toy in all directions: upward, downward, left, and right.

Due to feeding difficulties and poor weight gain at 3 years old, the patient underwent a percutaneous endoscopic gastrostomy (PEG). She remains on continuous feeding with PaediaSure Peptide. The feeding rate is currently 45 mls/h for 22 h per day which equals 990 mls Paediasure Peptide (990 kcals, 29.7 g protein). Despite this regimen, weight gain is still very slow, and she continues to exhibit failure to thrive—patient is at present 102 cm tall and weighs 12.2 kg kilograms (below the 0.4th percentile). The patient often experiences gastroesophageal reflux and constipation, requiring, respectively, omeprazole 15 mg once a day and lactulose 5–10 mL twice daily. At the age of 5, the patient underwent a cleft lip repair.

Serial cardiac evaluations demonstrated partial or complete spontaneous closure of the cardiac defects over time. Serial ECGs confirmed normal sinus rhythm, and echocardiography demonstrated normal biventricular function, myocardial structure, and chamber dimensions. The bicuspid aortic valve showed a normally sized aortic root, without significant stenosis or regurgitation, and forward flow velocity across the aortic valve remained within normal limits.

### 2.3. Current Condition

At the age of 6 years, nephrocalcinosis was detected by renal ultrasonography. In accordance with parental wishes, no further diagnostic workup to determine its etiology was pursued. Renal function tests, urine cultures, and serum electrolyte levels were all within normal limits. It was additionally confirmed that the patient received an appropriate intake of vitamin D_3_ (ranging from 1000 to 2000 IU daily).

The patient also presents disrupted sleep patterns. Melatonin 4 mg, administered three times per week, provides limited control over sleep dysregulation. Neuroimaging was proposed to the parents on several occasions; however, it would have required general anesthesia. Due to concerns about potential complications associated with anesthesia, the parents decided not to proceed with the examination. This decision aligned with core pediatric palliative care principles, including shared decision-making with the clinical team and prioritization of the child’s quality of life [13]. Given the patient’s baseline condition, neuroimaging was unlikely to alter her management, and proceeding would have primarily exposed her to procedural risks without clear benefit.

**Figure 2:**
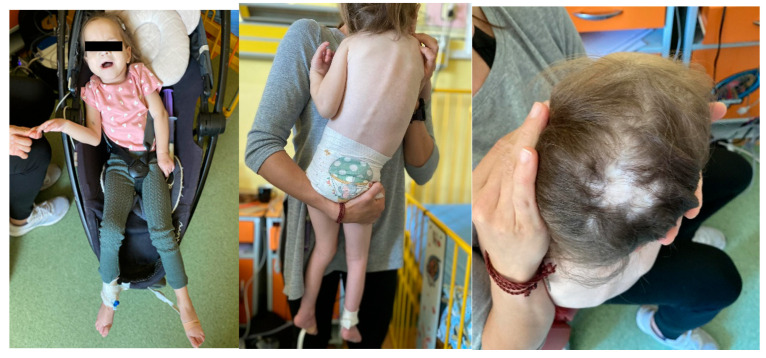
The 7-year-old female patient with Patau syndrome hospitalized due to pneumonia at Chair and Clinical Department of Pediatrics, Hospital No. 2 Bytom, School of Health Sciences, Medical University of Silesia in Katowice, Poland. Notable findings include failure to thrive, spinal deformity and aplasia cutis.

The most recent admission to the hospital was in October 2024 (Figure 2) due to pneumonia and lasted 10 days. She was treated with IV and oral antibiotics via her gastrostomy, required oxygen, but no ventilatory support. In the past, the girl was hospitalized multiple times due to pneumonia, with the longest interval between episodes lasting 2 years. She requires ongoing sputum and saliva suctioning daily, 0.9% saline nebulization as needed, and salbutamol inhaler spacer 2–4 puffs as required.

### 2.4. Individualized Approach

**Figure 3:**
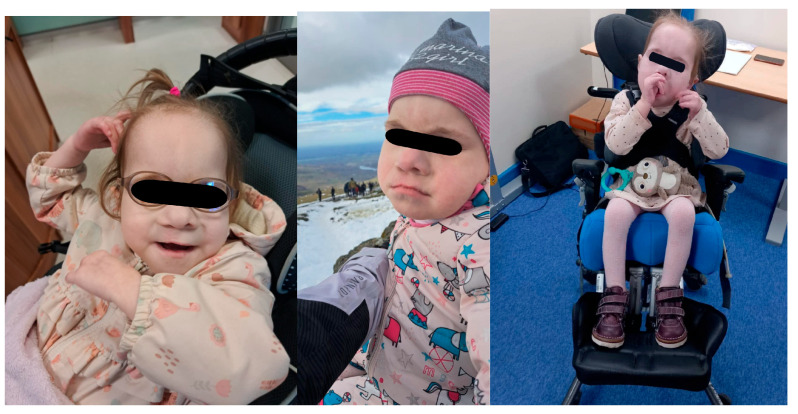
The female patient with Patau syndrome in her daily activities.

Despite the severity of the case, the patient, who is now 8 years old, receives a comprehensive, multidisciplinary approach, which likely contributes to her continued survival. The girl attends regular follow-ups with cardiologist, pulmonologist, audiologist, ophthalmologist, otolaryngologist, and gastroenterologist due to her underlying condition. Pharmacological treatment, medical adjustments and various therapies, including physiotherapy, rehabilitation that includes sensory sessions, speech and occupational therapies, have a positive impact on the girl’s daily life. The patient attends a primary school with specialist support that fosters her physical and sensory development and promotes progress in communication. Although the patient remains fully dependent for all mobility and self-care needs, she shows slow but constant improvement in her movement abilities. She can roll and reach to both sides, play with toys positioned above her chest, lift her head and arms when motivated and briefly maintain unsupported head control when held against gravity. She can sit with support and benefits from daily use of a standing frame. The patient accompanies her family’s mountain expeditions (Figure 3). The girl responds to speech with smiles and laughs, she enjoys watching her brothers play and cries when she wants to be cuddled; however, she has no significant speech development and has severe cognitive disabilities. Despite the numerous obstacles she faces, the patient’s journey illustrates the growth and development with the support of an interdisciplinary care team. It highlights the critical role of personalized care and early intervention.

## 3. Discussion

Limited information about the clinical course of patients surviving past infancy is available in the literature making it challenging to provide accurate counseling to parents following a new diagnosis. Due to the increased survival associated with better availability of care for trisomy 13, significant postnatal complications may present in patients with Trisomy 13 [9]. Patau syndrome is associated with a wide range of complications that may arise in these patients. Consequently, a comprehensive approach is essential throughout the patient’s life to optimize care and enhance quality of life. As we have observed, coordination among various specialists as well as repeated medical appointments are required. Survival time depends partly on the results of cytogenetic testing—whether it is full trisomy 13 or mosaic trisomy 13—and partly on the presence of severe somatic anomalies [14]. A population-based study from Japan recognized a cardiovascular-dominant subgroup of patients with trisomy 13 showing the highest early mortality (83%), supporting previous findings that congenital heart defects are major independent risk factors for death before one year old [15].

Our observations are consistent with the largest published cohort of long-term survivors with Trisomy 13, in which early-onset epilepsy was commonly reported. Similarly to our patient, seizure control was achieved in some individuals through chronic antiepileptic therapy. Feeding difficulties frequently necessitated gastrostomy placement in childhood, reflecting the long-term enteral nutrition required in our case due to persistent failure to thrive. Recurrent respiratory infections and sleep disturbances were also described in that cohort, highlighting the ongoing vulnerability of the respiratory system and the sustained caregiving burden on families. These features are likewise present in our patient, who requires regular airway clearance and has experienced multiple episodes of pneumonia. Despite profound global developmental impairment, preserved social responsiveness and affective engagement were noted in the cohort, consistent with our patient’s meaningful, though nonverbal, interaction with her environment and caregivers [11].

We believe that the long survival in our case is possibly due to supportive treatment, such as an omphalocele surgery in the first few days of life, gastrostomy, cleft lip surgery, antiepileptic medications, daily sputum and saliva suctioning and a well-organized home, although our patient had certain risk factors for early death, including preterm birth and congenital heart defects. Tailoring interventions to the patient’s evolving clinical needs in order to support her development at every stage of life is crucial. There is a study based in USA indicating that there was not a significant difference in mortality rate in Trisomy 13 patients that underwent more procedures in hospital setting compared to the ones that were discharged sooner [16].

It is important to understand that the prognosis of this disorder is typically poor and even though aggressive intervention may prolong median survival, not all pathologies can be addressed through conventional treatment approaches. For example, the timing of cleft lip and cleft palate repair is characterized by a tension between the need for early intervention to support speech development—typically between 9 and 12 months of age [17]. In patients with Patau syndrome (trisomy 13), a careful balance must be achieved between the risk of surgery and the potential benefits. Notably, a diagnosis of Trisomy 13 alone should not automatically preclude patients from surgical consideration [18]. The number of procedures performed in this population has increased over time; however, the risk associated with surgery must be weighed carefully. These patients often experience prolonged recovery periods, leading to extended intensive care unit (ICU) and hospital stay. In our patient, the cleft lip remained uncorrected until the age of 5, when the remaining comorbidities were well managed, in accordance with the child’s best interests. The cleft palate is not repaired.

An omphalocele, a relatively rare abdominal wall defect occurring in approximately 1 in 4000 live births [19], was surgically repaired in our patient on the third day of life due to its significant size >2 cm. According to published reports, performing surgical intervention within the first month of life in a child with Patau syndrome may be associated with an increased likelihood of survival beyond the first year of life [14].

Consideration should also be given to the burden of caring for a child with Patau syndrome, including not only psychological but also physical and financial aspects. Mental health specialists should be utilized as soon as the diagnosis is made, and participation in parental support groups may be beneficial for the parents and families [20]. The patient is unable to walk independently due to motor delays and musculoskeletal abnormalities. She also presents severe cognitive impairment and lacks speech capabilities. However, she appears to display positive emotional responses and facial expressions when interacting with her family, and cries when seeking affection. The use of hearing aids may have contributed to improved cognitive development. Another ongoing concern is sleep disturbance, which is commonly observed in children with Patau syndrome [21,22]. The patient exhibited nocturnal sleep disturbances, which negatively affected not only her own sleep quality but also that of her family—particularly her parents, who serve as her primary caregivers.

The ultrasound findings in our patient may be suggestive of nephrocalcinosis; however, this diagnosis requires confirmation through additional diagnostic methods, such as low-dose non-contrast CT or, in selected cases, endoscopic evaluation [23]. It has been reported that nephrocalcinosis may regress or resolve over time [24]. So, in our case, regular follow-up with renal ultrasonography is recommended.

A study conducted at a quaternary university hospital in São Paulo demonstrated that infants who received Pediatric Palliative Care (PPC) were often discharged home, highlighting the importance of initiating this type of support early—not solely at the end of life. PPC is a specialized medical care that should be indicated as soon as a life-limiting condition is diagnosed, whether there is availability or not of curative treatment [25].

### Clinical Implications

Our case report highlights several key clinical lessons for practitioners caring for patients with Trisomy 13:

- Comprehensive and ethically sensitive counseling is crucial when communicating a prenatal or postnatal diagnosis of Trisomy 13. Clinicians should present evidence-based prognostic data and evolving management options in a balanced manner, facilitating shared decision-making with families.
- Individualized surgical decision-making is essential. Procedures such as omphalocele repair or cleft lip correction should be considered based on the patient’s overall stability, comorbidities, and family preferences, rather than the chromosomal diagnosis alone.
- Early incorporation of palliative and psychological care, combined with systematic follow-up for potential late-onset complications, is fundamental for optimizing both patient outcomes and family well-being.

We believe our study will provide valuable insights into the living conditions and psychomotor development of long-term Patau syndrome survivors.

## 4. Conclusions

Our case report demonstrates that considering Patau syndrome as universally fatal is no longer appropriate. Potential long-term survival should be acknowledged, and proactive management strategies should be implemented from an early stage. Furthermore, we see that it is important to provide medical and social services to reduce the burden on families.

Finally, multicenter studies are needed to better characterize the clinical course of long-term survivors and to develop standardized care protocols aimed at improving quality of life for both patients and their families. In patients requiring chronic pediatric care, it is worth introducing and improving techniques that allow for an objective assessment of the complexity of nursing care to facilitate the organization of patient care, hospital resource management and cooperation between the interdisciplinary team [26,27].

## Data Availability

The data that support the findings of this study are not publicly available due to patient confidentiality and privacy concerns but can be provided by the corresponding author upon reasonable request.

## Abbreviations

The following abbreviations are used in this manuscript:

NICU	Neonatal Intensive Care Unit
VSD	Ventricular Septal Defect
ASD	Atrial Septal Defect
PFO	Patent Foramen Ovale
ABR	Auditory Brainstem Responses
PEG	Percutaneous Endoscopic Gastrostomy
ECG	Electrocardiography
IU	International Unit
ICU	Intensive Care Unit
CT	Computed Tomography
PPC	Pediatric Palliative Care PPC
